# Synthesis and catalytic applications of combined zeolitic/mesoporous materials

**DOI:** 10.3762/bjnano.2.87

**Published:** 2011-11-30

**Authors:** Jarian Vernimmen, Vera Meynen, Pegie Cool

**Affiliations:** 1 Laboratory of Adsorption and Catalysis, Department of Chemistry, University of Antwerp, Universiteitsplein 1, B-2610 Wilrijk, Belgium

**Keywords:** catalysis, characterization, combined zeolitic/mesoporous materials, synthesis

## Abstract

In the last decade, research concerning nanoporous siliceous materials has been focused on mesoporous materials with intrinsic zeolitic features. These materials are thought to be superior, because they are able to combine (i) the enhanced diffusion and accessibility for larger molecules and viscous fluids typical of mesoporous materials with (ii) the remarkable stability, catalytic activity and selectivity of zeolites. This review gives an overview of the state of the art concerning combined zeolitic/mesoporous materials. Focus is put on the synthesis and the applications of the combined zeolitic/mesoporous materials. The different synthesis approaches and formation mechanisms leading to these materials are comprehensively discussed and compared. Moreover, Ti-containing nanoporous materials as redox catalysts are discussed to illustrate a potential implementation of combined zeolitic/mesoporous materials.

## Introduction

Nanoporous materials are characterized by their relatively high surface areas and pore volumes within a small amount of material. These properties, together with the fact that they have (uniform) channels and voids in the nanometer range, make them ideal candidates for implementation in several applications. In fact, nanoporous materials are used extensively in a wide variety of applications on industrial, pilot, and laboratory scale in many different research areas, such as fine and specialty chemistry [1–3], petrochemistry [4–5] and medicine [2,6–10]. They can be applied as catalysts [1–5,11], drying agents [5,12], adsorbers [5,13], sensors [14–15], controlled-drug-release agents [6,8], column-packing material [16], food additives [17], etc. According to IUPAC (International Union of Pure and Applied Chemistry) nomenclature, nanoporous materials are classified in categories of microporous (pore diameter <2 nm), mesoporous (pore diameter 2–50 nm) and macroporous (pore diameter >50 nm) structures. The enormous diversity in nanoporous structures as well as the fact that their properties can be tuned and modified depending on the type of application is responsible for the huge interest in these materials among different scientific communities. Therefore, research has been focused on the elucidation of the formation mechanism, the development of new, tailor-made nanoporous structures, and the implementation of the materials in various processes and applications. More specifically, in the last decade, the development of mesoporous materials with zeolitic features has received a lot of attention. These combined zeolitic/mesoporous materials are thought to be superior materials, since they are able to combine (i) the enhanced diffusion and accessibility for larger molecules and viscous fluids of mesoporous materials with (ii) the remarkable stability, catalytic activity and selectivity of zeolites.

This review gives an overview of the state of the art in the development of combined zeolitic/mesoporous materials. It is divided into two parts. In the first section, the synthesis methods and formation mechanisms of the combined zeolitic/mesoporous materials are described. In addition, a thorough evaluation of the different synthesis strategies leading towards combined zeolitic/mesoporous materials is carried out, in which their advantages and disadvantages are discussed and a comparison is drawn between the different methods. In the second part, Ti-containing nanoporous materials as redox catalysts are used as an example to illustrate the potential implementation of combined zeolitic/mesoporous materials. Although there is a huge variety of combined zeolitic/mesoporous materials with deviating properties (sorbent, acidic, redox, basic), the examples throughout this review, including the section on the implementation of combined zeolitic/mesoporous materials, are specifically focused on Ti-containing siliceous materials. This is because our research group has much experience with these types of materials. Moreover, the different aspects we want to highlight during this review can be perfectly demonstrated with Ti-containing combined zeolitic/mesoporous materials. Despite the focus on Ti-containing combined zeolitic/mesoporous materials, we are convinced that the conclusions and observations in this review are valid for the majority of combined zeolitic/mesoporous materials, irrespective of the active element.

## Review

### 1 Combined zeolitic/mesoporous materials

#### 1.1 Evolution towards combined zeolitic/mesoporous materials

The large-scale implementation of zeolites in industrial applications and the still-growing amount of publications involving zeolites prove that these materials are of prime importance in a wide variety of scientific fields. This is without a doubt due to their crystalline, microporous 3-D structure and their ability to accommodate many different heteroelements (other than Si and O). Their remarkable stability (mechanical, hydrothermal, thermal and chemical) and high catalytic activity and (shape) selectivity make zeolites unique materials. However, despite these wonderful properties, zeolites have one major drawback: Their microporous nature causes accessibility problems and diffusion limitations for large molecules and viscous fluids [2,18]. In the specific case of TS-1 [19], only molecules with a kinetic diameter of maximally 0.6 nm (e.g., benzene) can access the structure and reach the active sites. This drastically limits their implementation in, e.g., fine and specialty chemistry, pharmaceutical industry and biological applications, for which large, bulky molecules are often required. Possible solutions for this limitation are (i) to decrease the crystal sizes of the zeolites and/or (ii) to develop materials with larger pores [20]. The first option was applied to reduce the intracrystalline diffusion path length [21]. However, separation of these nanozeolites is difficult, since they tend to aggregate and form colloidal solutions. Moreover, nanozeolites often have different properties compared to their larger counterparts, such as a diminished crystallinity, resulting in a loss of catalytic activity and lower stability [20–21]. The second option is the creation of nanoporous materials with larger pores, namely mesoporous materials, such as SBA-15 [22–23], SBA-16 [23], M41S [24–26], MSU [27–31], MCF [32] and many others [33–34]. These mesoporous materials can overcome diffusion and accessibility problems, and this has opened up new perspectives in, for example, catalysis and medical applications. More detailed information on the formation mechanisms, the applications and the characteristics of mesoporous materials can be found in several excellent reviews [33–37]. Although there is large diversity in structural properties among mesoporous materials, these materials all have in common that their (metallo)silicate framework is not crystalline, but amorphous. This implies that their stability is inherently lower than that of zeolites [38]. Moreover, their amorphous nature and specific synthesis conditions often cause difficulties for the incorporation of heteroelements into their structure, which results in a lower catalytic activity than in the case of zeolites. For example, due to the harsh acidic medium that is required for the synthesis of SBA-15, leaching of heteroelements such as Ti is unavoidable during in situ syntheses, limiting their catalytic activity [39–42]. Moreover, in some occasions the amorphous siliceous framework hampers the solid incorporation of heteroelements in the specific coordination needed for catalysis. For example, the incorporation of Ti in tetrahedral positions, as in TS-1 zeolites, is difficult in Ti-MCM-41 since the structure contains a lot of defects, resulting in the formation of a substantial amount of octahedrally coordinated Ti [35,43].

**Figure 1:**
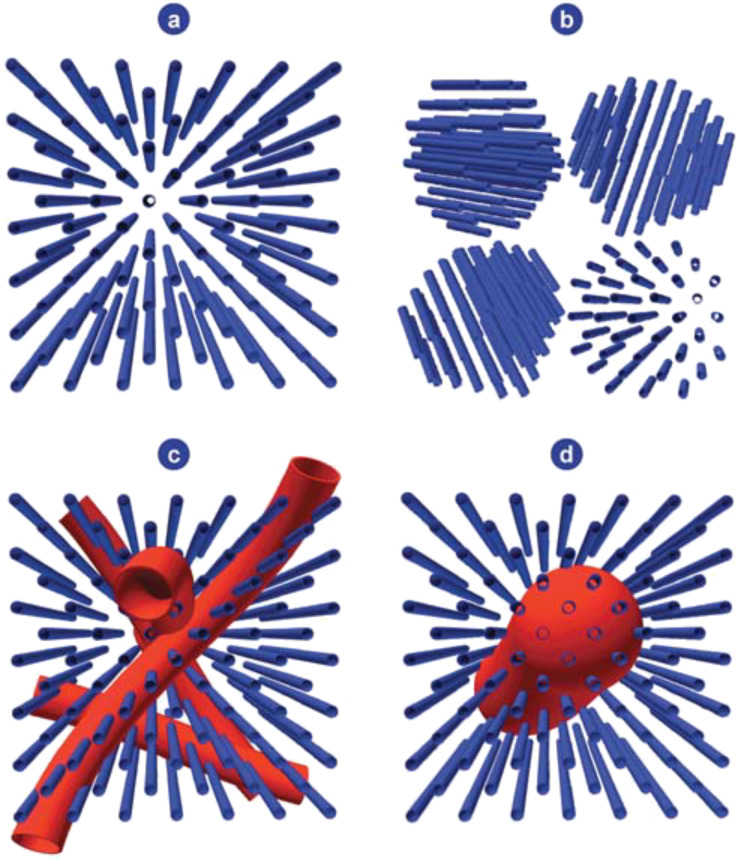
Different degrees and types of hierarchy can be defined in porous materials [48]. Reproduced by permission of The Royal Society of Chemistry.

Therefore, in the last decade, research has been focused on the combination of mesoporosity and zeolitic features [20,44–50]. Many promising materials, such as MTS-9 [51] and Ti-MMM-1 [52] have already been developed. The ultimate goal is to develop a “true” hierarchical mesoporous zeolite, meaning a mesoporous material with zeolitic walls, wherein the micro- and mesopores are interconnected in order to form a hierarchical structure. Such mesoporous zeolites are expected to be superior materials since they will be able to catalyze the typical reactions in which standard zeolites are used, but instead of being limited by the microporous nature, they will be able to convert larger molecules as well [44]. Moreover, the presence of a hierarchical pore system is also considered to be beneficial, since the presence of two interconnected pore systems with different dimensions ensures (i) a high mass transfer through the mesopores; (ii) a high surface area available for interaction of the molecules with the active sites and (iii) the catalytic features (i.e., shape selectivity) of the micropores [44–45]. However, a proper connectivity between the various levels of pores is vital to maximize the benefit of hierarchy in catalyzed reactions. J. Pérez-Ramírez et al. [48] discussed the different degrees of hierarchy that can be obtained in a combined zeolitic/mesoporous material (Figure 1). In Figure 1a, the reference zeolite system is depicted, which gives rise to mass transfer and accessibility problems for large molecules. In Figure 1b, four nanozeolites are bridged by interparticle mesoporous voids. In Figure 1c and Figure 1d on the other hand, “real” mesopores exist in combination with micropores, but in the case of Figure 1d the mesopores are only accessible via the micropores. Here, it is clear that not all configurations with a combined micro- and mesoporosity inherently give rise to an enhanced molecular transport to or from the active sites in the micropores. However, this does not mean that these combined zeolitic/mesoporous materials without (or with only minor) “true” hierarchical ordering cannot be beneficial for certain reactions. In fact, often a uniform pore size distribution and a high level of porosity are much more relevant than a perfectly ordered nanoporous material. The disordered mesoporous material, KIT-1 [53], is the perfect example illustrating that the absence of any ordering does not necessarily imply that the material cannot be useful, and in fact it may be even better than its ordered analog (MCM-41) in certain cases [54–56]. Considering combined zeolitic/mesoporous materials, there are several examples of materials that give rise to an enhanced catalytic performance in comparison with their purely zeolitic and/or mesoporous counterparts [51–52,57–59]. MTS-9 for example [51], gives rise to a higher catalytic activity in the epoxidation of styrene than Ti-MCM-41 and shows a selectivity and activity similar to TS-1 zeolite. In the hydroxylation of 2,3,6-trimethylphenol, MTS-9 is more active than both Ti-MCM-41 and TS-1. However, there are also examples of combined zeolitic/mesoporous materials, wherein there is no significant improvement when a comparison is drawn with standard zeolites or mesoporous materials [60–62]. Cheneviere et al. [60] showed that their developed mesoporous TS-1 material does not give rise to the expected improved catalytic properties of a hierarchical catalyst in oxidation reactions with aqueous H_2_O_2_, probably because of its increase in hydrophilic character in comparison with a conventional zeolite. Thus, whether a combined zeolitic/mesoporous material shows great potential or not, does not necessarily depend only on its “true” hierarchical character. The type of application as well as the structural characteristics of the material, and therefore also the synthesis method, are without a doubt equally important aspects.

#### 1.2 Synthesis of combined zeolitic/mesoporous materials

Table 1 gives an overview of the most important synthesis approaches to obtain combined zeolitic/mesoporous materials [44–49]. There also exist other, more exotic approaches, such as nuclear track imprinting [63], but since their use is not very widespread, they will not be dealt with here. The synthesis methods can be roughly divided into three different classes, namely postsynthetic, templating and nontemplating approaches. In the postsynthetic approach, a zeolite or mesoporous material is first formed before being subjected to an additional treatment. The main difference between templating and nontemplating synthesis strategies is whether or not a mesotemplate is used in order to create combined zeolitic/mesoporous materials (the classification is analogous to purely mesoporous materials) [44]. Note that the majority of the templating and nontemplating methods listed in Table 1 are exactly the same as in the synthesis of purely mesoporous materials, meaning that these approaches have simply been extrapolated to the synthesis of combined zeolitic/mesoporous materials by replacing the silica source with zeolitic nanoparticles (two-pot templating approach). In addition, there are also unique approaches that have been specifically developed for the formation of combined zeolitic/mesoporous materials, such as the one-pot templating synthesis in which the micro- and mesotemplate are added to the same reaction vessel.

**Table 1:** Overview of the most important synthesis strategies for the formation of combined zeolitic/mesoporous materials.

postsynthetic	templating		nontemplating

demetallation	**hard**^a^	**soft**^a^	mesotemplate-free synthesis (sol–gel)
recrystallization	carbon	MOS^b^: one-pot, two-pot	self-formation mechanism of hierarchy
deposition	aerogel, polymer, resin	POS^c^: one-pot, two-pot	
delamination	biological materials		

^a^The classification of “hard” (or solid or textural) and “soft” templates is based on the physical nature of the mesotemplates. ^b^MOS: molecular organized systems; ^c^POS: polymeric organized systems.

An alternative classification of the different synthesis approaches that is often applied is the distinction between the bottom-up and top-down synthesis strategies [48]. On the one hand, the bottom-up methods build the materials from the precursors up, meaning that they start from building units and chemicals in order to constructively form the combined zeolitic/mesoporous materials. On the other hand, the top-down approaches are focused on the controlled removal of material from an already existing structure in order to create combined zeolitic/mesoporous materials. In the subsequent part, the different methods (listed in Table 1) along with their advantages and disadvantages, will be discussed. The examples will be mainly focused on Ti-containing combined zeolitic/mesoporous materials.

#### 1.2.1 Postsynthetic approach

**Demetallation:** In demetallation, a metal is selectively removed from the framework of a zeolite by postsynthetic steaming, chemical treatment, or acid or base leaching, resulting in randomly created voids in the mesoporous range [45,50]. The oldest form of demetallation and at the same time the first technique applied for creating mesopores in zeolites is dealumination [64]. By subjecting Al-containing zeolites to a hydrothermal treatment (steaming) and/or acid leaching, the Si–O–Al bonds are hydrolyzed, resulting in a partial destruction of the silicate framework. The dealumination process occurs randomly and depends highly on the amount of Al incorporated in the structure and on the applied extraction method. Although the created mesopores are beneficial and dealumination is a simple, widely used (industrial) procedure, the main drawbacks [44–45,50] of this method are (i) the partial amorphization of the zeolite framework; (ii) the loss of catalytic activity as part of the active element is removed; (iii) the fact that the mesopores are rather cavities that are not interconnected to form a mesoporous network; (iv) the random nature of the mesopore formation; (v) the fact that the porosity of dealuminated zeolites is seriously altered in an uncontrolled way during regeneration at high temperature; (vi) the partial blockage of the active sites by deposition of amorphous material inside the meso- and micropores and (vii) the restriction to Al-containing zeolites.

**Figure 2:**
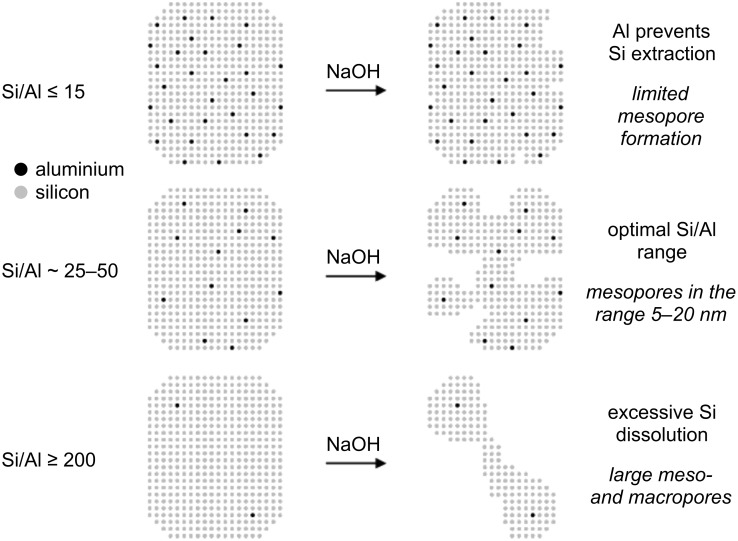
A simplified representation of the influence of the Al content on the desilication of ZSM-5. Reprinted with permission from [66]. Copyright 2004 American Chemical Society.

A very promising alternative and highly reproducible method is desilication [48,50,65–67]. Extraction of Si atoms by base treatments (also denoted as pore-directing agents, PDAs) leads to a significant amount of intracrystalline mesoporosity while preserving the intrinsic acidity and structural integrity of the zeolite framework. Also during this process, Al present in the zeolite framework plays a key role: For the ZSM-5 zeolite, Groen et al. demonstrated that the optimal Si/Al ratio is 25–30 (Figure 2) [66]. However, the fact that Al needs to be present in the framework immediately exposes the drawback of this synthesis approach, namely that the desilication is limited by the initial Si/Al framework. A good alternative is the combination of dealumination and desilication, since this allows an extra flexibility regarding the Si/Al ratios and a decoupled modification of mesoporous and acidic features [65]. Moreover, recently Verboekend et al. [68] showed that by complementing the alkaline solution with external (large size) PDAs full compositional flexibility can be achieved in the preparation of mesoporous zeolites by the desilication method. By proper control of the synthesis conditions, even Al-free zeolites can undergo a successful desilication treatment. A few studies on detitanation [69–70] and deboronation [71–72] have also been reported.

**Recrystallization:** Recrystallization is a typical top-down approach since it starts from a full-grown zeolite or a purely mesoporous structure, which is then impregnated by either a mesotemplate [73–74] or a microtemplate [75–76], and subjected to a hydrothermal treatment. This results in dissolution of the silica, which will then interact with the template and recrystallize in a mesoporous or zeolitic phase depending on the added template. The degree of crystallinity or mesoporosity can be altered by changing the reaction conditions. In general, recrystallization will give rise to composite zeolitic/mesoporous materials rather than true hierarchical materials [50]. In the case of recrystallization of a mesoporous material, it is important to start from a structure with thick walls (such as SBA-15) or to fill the mesopores with a support material (such as the mesotemplate [77]) in order to prevent a collapse of the mesopores during zeolitization. An example of a combined zeolitic/mesoporous material synthesized by the recrystallization route is UL-TS-1, which is formed by heating TPAOH-impregnated, amorphous, Ti-containing mesoporous materials for several days at 120 °C [78–79].

**Postsynthetic deposition of zeolitic nanoparticles into mesoporous materials:** In this method, a presynthesized mesoporous material is impregnated with a zeolite precursor solution [44,79–85]. This solution is obtained by terminating the zeolite formation in an early stage, often before the hydrothermal treatment, so that the zeolitic nanoparticles cannot evolve into a full-grown zeolite [51]. For MFI zeolites, the nanoparticles size is typically around 4 nm, although there is no consensus on the shape of the particles [86–88]. The mesoporous supports are generally large-pore materials, such as SBA-15 and MCF (mesocellular foam) so that the nanoparticles can be accommodated inside the mesopores. The impregnation of the nanoparticles can occur through wet, incipient wetness and dry impregnation. During a wet impregnation, the mesoporous material is completely soaked in a solution of nanoparticles, while in the case of a dry impregnation a volume of solution identical to, or even smaller than, the total pore volume is added. An incipient wetness impregnation lies between these two extremes.

**Figure 3:**
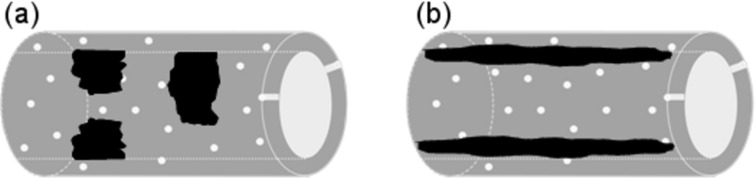
Schematic representation of (a) a plugged and (b) a coated material, obtained by the postsynthetic deposition of zeolite nanoparticles into SBA-15. Reprinted with permission from [85]. Copyright 2011 American Chemical Society.

By applying this synthesis strategy, a combined zeolitic/mesoporous material is obtained with zeolite-like (microporous) nanoplugs and/or a zeolite-like coating (inside the pores and/or on the outside of the material). In the case of a mesoporous material with zeolitic nanoplugs in the mesopores, a plugged hexagonal templated [89–90] (PHTS)-like material can be formed (Figure 3a) [85]. PHTS is a mesoporous material, which is obtained by increasing the silica/surfactant ratio in the SBA-15 synthesis. The excess amount of silica source gives rise to the formation of amorphous microporous plugs inside the mesochannels of SBA-15, resulting in a PHTS material with both open and narrowed pores. In the case of a coating inside the pores of the mesoporous support, a decrease in the mesopore diameter is observed (Figure 3b) [85]. Notice that this synthesis approach not only leads to the formation of a combined zeolitic/mesoporous material, but also enables the study of the zeolite nanoparticles. A drawback of this method is that the active sites are only located in the coating/plugs and not throughout the mesoporous structure itself [85]. In addition, as previously mentioned, the small dimensions of the nanoparticles often alter the properties of the materials as compared to the full-grown zeolite (e.g., hydrophilicity) [83,85,91]. Nevertheless, examples of beneficial materials obtained by the postsynthetic deposition approach also exist, namely the TS-1 coated MCF structure developed by D. Trong-On [79,82], which shows a higher activity for the oxidation of 1-naphthol than the mesoporous Ti-MCF material and the TS-1 zeolite.

**Figure 4:**
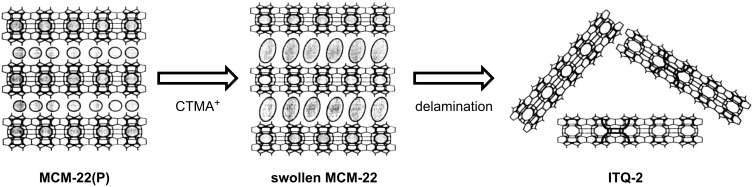
Schematic representation of the delamination process (CTMA^+^ = cetyltrimethylammonium ion). Reprinted from [93]. Copyright 2000, with permission from Elsevier.

**Delamination:** This technique is only applicable for those zeolites (i.e., MCM-22 and ferrierite) that have lamellar structures (Figure 4) [47,92–94]. First, the as synthesized zeolite precursor [MCM-22(P)] (with the microtemplate still in the structure) is allowed to interact with a surfactant, resulting in a swollen, intercalated structure. After removal of the surfactant, the intercalated structure is exfoliated and collapses to form a highly mesoporous material made of randomly packed zeolite sheets with the microporous structure of the parent zeolite [ITQ-2] preserved. Here, all active sites are directly accessible from the external surface, but only a limited control over the resulting mesopore size is possible. Ti-containing ITQ-2 structures can be obtained by grafting of titanocene complexes. These turn out to be excellent catalysts for the epoxidation of olefins [95].

#### 1.2.2 Templating approach

In templating approaches, combined zeolitic/mesoporous materials are obtained by using appropriate templates. Strictly speaking, the term “templating approach” relates to the mesotemplate and not to the microtemplate. This is because in all the templating strategies, only the type of mesotemplate is changed and not the microtemplate.

In general, a distinction is made between hard (or solid, or textural) and soft templates, which reflects the physical nature of the mesotemplates, analogous to the distinction that is made in the syntheses of the purely mesoporous materials. The most frequently used hard templates are carbon-based, biological and polymeric ones, whereas the soft templates are the typical molecular organized systems (MOS) and polymeric organized systems (POS), mostly identical to the surfactants applied for the formation of purely mesoporous materials. Other surfactants are also being developed specifically for their beneficial influence on the formation of mesoporous materials with zeolitic character.

**Hard templating route:** Here, the zeolite is grown in the presence of a solid material. First, a solution of zeolite nanoparticles (precursor solution of a full-grown zeolite: See postsynthetic deposition) is prepared, before being combined with a hard template. Then, the mixture is subjected to a hydrothermal treatment so that the zeolite can be formed in or around the solid “mould”. Finally, the zeolite structure-directing agent as well as the hard template are removed, resulting in the formation of a mesoporous zeolite with a structure that is fully determined by the morphology of the hard template and the zeolite [20,47–48,50].

**Figure 5:**
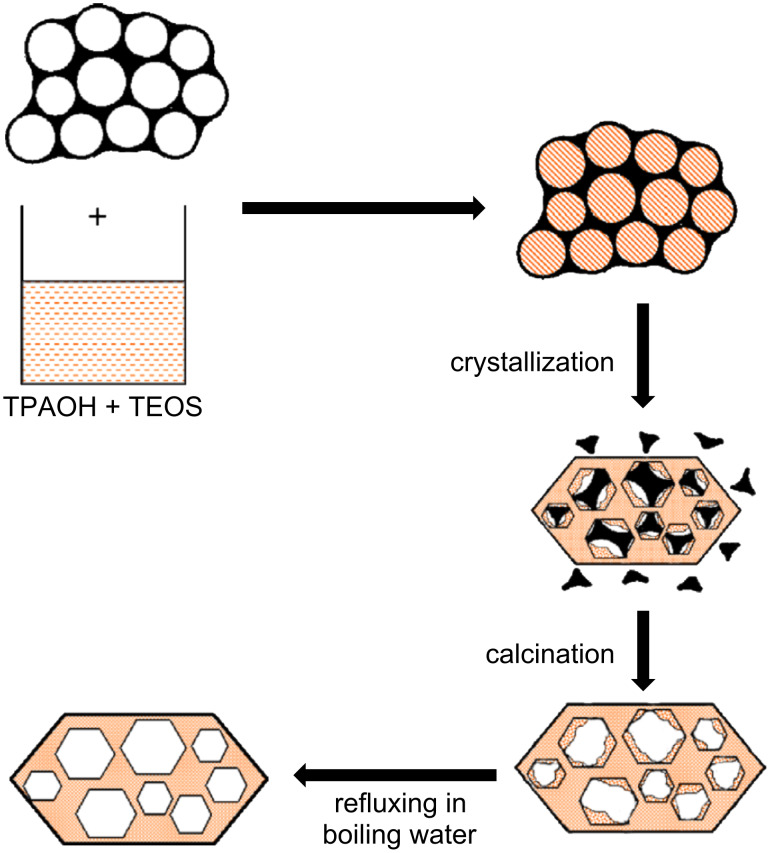
Schematic representation of the carbon-based hard-templating route for the formation of mesoporous silicalite-1 (MFI) zeolite. In the first step, the zeolite precursor solution is combined with a mesoporous carbon template (= black matrix). Then the zeolite is crystallized and the crystals entirely or partially encapsulate the porous carbon. In addition, amorphous silica is also formed. The carbon matrix is removed by calcination. Finally, the amorphous silica is dissolved by refluxing the material in boiling water, resulting in the formation of mesoporous silicalite-1. Reprinted from [98]. Copyright 2007, with permission from Elsevier.

Carbon-based templates are the most common type of hard template. The concept of using carbon in combination with zeolites was first applied to obtain nanosized zeolites in the so-called confined-space synthesis, whereby zeolites were grown inside the voids of porous carbon [47,96]. However, by altering the synthesis conditions, it is also possible to completely encapsulate the porous carbon matrix [97], resulting in the formation of mesoporous zeolite crystals (Figure 5) [98]. A wide variety of carbon templates can be used, such as carbon black [99–101], ordered mesoporous carbons (CMKs) [102–103], carbon nanotubes [104], and carbon nanofibers [105]. The choice of template is crucial for the final material, since the mesoporous zeolites essentially become replicas of the carbon pore system in which they grow. For example, with carbon black, the resulting pores will be nonuniform with no interconnection. However, this does not mean that these materials are not useful: Park et al. [101] described the use of carbon-templated mesoporous TS-1 for the epoxidation of cyclic olefines. Carbon nanotubes, on the other hand, give rise to uniform, straight mesopores, but are a more expensive alternative. The most promising, but also most expensive, carbon templates are the CMKs. These carbons are replicas or inverse replicas of existing mesoporous silicates, such as MCM-48 (CMK-1) [106] and SBA-15 (CMK-3 [107] and CMK-5 [108]). By impregnation of the CMKs with zeolite nanoparticles, replicated mesoporous materials (RMMs) are formed with a tunable degree of zeolitic character and a wide choice in mesoporosity [102]. This synthesis strategy is very appealing, since it is applicable to practically all zeolites and offers a fairly good control over the porosity. However, it is an expensive technique with potential health, safety and environmental issues, because of the high production costs and the need for extensive combustion to remove the carbon [50].

Aerogel, polymer, resin and biological templates can also be used as hard templates [20,47], although the number of publications is lower than for carbon-templated combined zeolitic/mesoporous materials. Carbonized as well as noncarbonized resorcinol–formaldehyde (RF) aerogels can be applied [109–112]. They both have a tunable degree of porosity, for example, by altering the RF-ratio. Nevertheless, the noncarbonized RF aerogels are inherently less porous than the carbonized ones. Another option is to use polystyrene [113], latex [114] or resin spheres or beads [115], although they are mostly used for macrotemplating. Also biological templates, such as starch [116], bacterial threads [117], wood cells [118], leaves and stems of plants [119], have been put forward as relatively inexpensive and abundantly available templates.

**Soft templating route:** When synthesizing combined zeolitic/mesoporous materials by the soft templating route, mesopores are formed by using, in most cases, the same surfactants (MOS/POS) as for regular mesoporous materials [33]. The large difference with the standard synthesis of mesoporous materials is that zeolite nanoparticles (zeolite precursor solution) are used as the silica (and heteroelement) source [44,47–48]. The goal is that the nanoparticles organize themselves around the surfactant assemblies and that they form the zeolitic walls of the resulting combined zeolitic/mesoporous material. A distinction is made between one-pot [52,57,120–122] and two-pot [51,123–135] templating strategies. In the latter case, the zeolite nanoparticles are formed beforehand in a separate reaction vessel. Subsequently, these nanoparticles are added to a mesotemplate solution, hence the term “two-pot”. In a one-pot synthesis on the other hand, the micro- and mesotemplates, together with the silica (and heteroelement) source, are all added to the same reaction vessel (at the same time). In this way, the mesopores and zeolite formation is intended to occur in one pot and ideally simultaneously. An example of a successful two-pot synthesis is the formation of MTS-9, a mesoporous titanosilicate with primary and secondary building units similar to TS-1 [51], and which shows a strong oxidation ability in the (substituted-) phenol hydroxylation and exhibits a high hydrothermal stability. Also one-pot templating approaches have already given rise to promising materials, such as Ti-MMM-1 [52], which is a much more selective catalyst for the oxidation of octane and cyclohexane than are Ti-MCM-41 or TS-1. Although various highly active materials have been developed through the soft templating method, in practice it is not clear whether the resulting materials are true hierarchical mesoporous zeolites. Indeed, unambiguous proof regarding this matter is often not supplied. In one of our papers [136], we demonstrated that it is not possible to obtain a true hierarchical mesoporous zeolite by applying a simple one-pot templating synthesis strategy, based on a TS-1 recipe. Here, part of the microtemplate (tetrapropylammoniumhydroxide) was replaced by a standard mesotemplate (cetyltrimethylammoniumbromide). We showed that because of the inherent competition between zeolite and mesopore formation, the creation of a mesoporous zeolite is inhibited. However, although no true hierarchical structure can be obtained, optimizing the synthesis parameters does lead to the formation of a combined zeolitic/mesoporous material with a pronounced zeolitic character and a high mesoporosity, denoted as meso-TSM [136–137]. This material can be used as redox catalyst in the epoxidation of cyclohexene [85].

**Figure 6:**
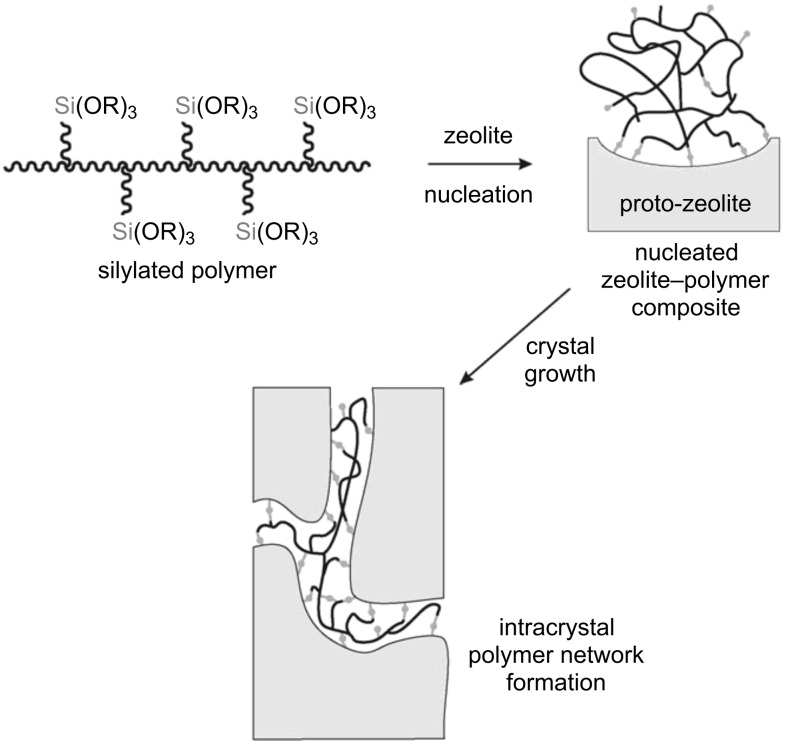
Conceptional approach for the synthesis of a mesoporous zeolite with a silylated surfactant as mesotemplate. Reprinted from [141]. Copyright 2006, with permission from John Wiley and Sons.

An advanced one-pot templating synthesis that does guarantee the formation of a true hierarchical mesoporous zeolite [138–141] is the use of tailor-made organic–inorganic hybrid surfactants. Here, a silylated surfactant is applied in combination with a synthesis gel with the composition of a zeolite. The covalent bonding between the zeolite precursors and the organosilane surfactant avoids the expelling of the surfactant-based mesostructure out of the crystallization of the zeolite phase during the synthesis (Figure 6). The number of publications on these tailor-made surfactants keeps on rising [60,142–146]. Recently, Ryoo and coworkers [143–146] developed new types of bifunctional surfactants (di- and polyquaternary ammonium surfactants) that combine the functionalities of a mesotemplate with those of a zeolite structure-directing agent. In this way, a single surfactant is able to direct the formation of mesoporous zeolites. The main drawback of this method is the use of non-commercially available, exotic and therefore expensive surfactants.

#### 1.2.3 Nontemplating approach

Since the price of mesotemplates is quite high (Table 2) [50] and their use gives rise to possible environmental risks, researchers have also explored the development of combined zeolitic/mesoporous materials by nontemplating approaches [147–154]. More specifically, these approaches are in the absence of templates for the mesopores, but the zeolite structure-directing agent is still used.

**Table 2:** Prices of templates used for the synthesis of nanoporous materials [50].

template	price per kg template (€)	price per kg final material (€)

CTMABr	530	387
P123	100	55
carbon nanofibers	2520	1814
carbon particles	6150	4551

**Figure 7:**
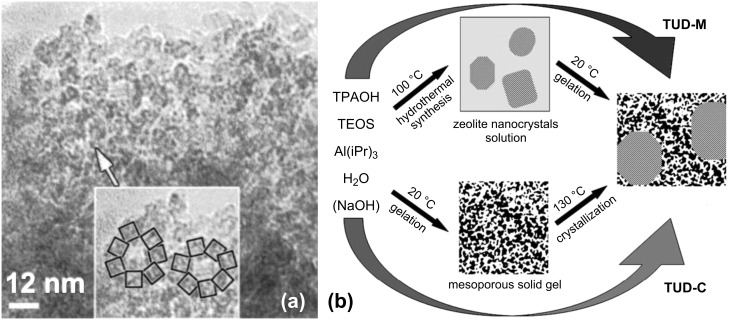
(a) Edge-sharing principle in the template-free synthesis in acidic medium [147] and (b) schematic representation of the TUD-C and TUD-M synthesis [152]. Reprinted from [147,152]. Copyright 2008 and 2009, with permission from Elsevier.

**Mesotemplate-free synthesis:** In 2008, Stevens et al. [147] reported the formation of a mesoporous material by acidic hydrothermal assembly of silicalite-1 nanoparticles in the absence of a mesotemplate. The crucial step in this synthesis is the acidification of the solution containing the zeolite precursor, which results in a ligand loss of the microtemplate [83]. In this way, the nanoparticles are not able to develop into a full-grown zeolite. Instead, they form assemblies by edge-sharing (Figure 7a), similar to the case in sol–gel synthesis [155], resulting in interparticle mesoporosity. The drawback in this synthesis approach is that it is a random process, which means that a proper control of the porosity is rather difficult and ordering of the mesopores is not possible. Moreover, in the specific case of silicalite-1, the resulting material does not show any long-range zeolitic character [147]. However, when using Beta nanoparticles [148], it is possible to obtain a combined zeolitic/mesoporous material with pronounced zeolitic features, although not as a “true” hierarchical system.

Another example of a template-free synthesis is the formation of TUD-C and TUD-M materials [149–152] (Figure 7b). The synthesis is also executed with zeolite nanoparticles, but in a basic medium instead of under acidic conditions. Here, the microtemplate will not only direct the micropore formation, but will also act as a scaffolding agent enabling the creation of a material combining zeolitic domains with a disordered amorphous mesoporous matrix. An advantage of this mesotemplate-free method in comparison with the former one is that leaching of the heteroelements (e.g., Al and Ti) will occur to a minor extent due to the basic synthesis conditions.

**Figure 8:**
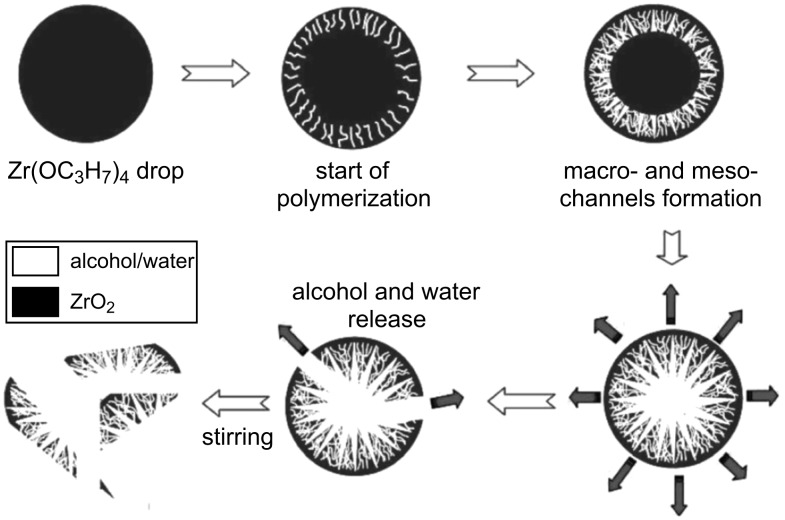
Schematic representation of the formation of micro-meso-macroporous metal oxides (zirconia) [154]. Reproduced by permission of The Royal Society of Chemistry.

**Self-formation mechanism of hierarchy:** The phenomenon of self-formation of porosity was first seen in the synthesis of metal oxides starting from metal alkoxides in water droplets (Figure 8) [154,156]. Here, the hydrolysis and condensation induce the formation of small molecules, namely water and alcohol, which then create porosity in a random manner. Recently Su and coworkers [153–154] extended this method to the synthesis of zeolites (TS-1; Beta; ZSM-5), whereby amorphous metaloxides are impregnated with microtemplates. This results in the formation of combined micro-, meso- and macroporous materials with zeolitic features. Since this approach is very new, the exact formation mechanism and detailed structural properties of the materials need to be explored in more detail before drawing any conclusion on its applicability.

#### 1.2.4 Conclusion

As pointed out in the preceding paragraphs, each synthesis strategy for the formation of combined zeolitic/mesoporous materials has its advantages and disadvantages. Table 3 summarizes the most important characteristics of the different approaches. The choice of which synthesis strategy is the most suitable one should always depend on the type of application that is being aimed at and on the resources available. A trade-off needs to be made between these two aspects in order to decide which route to follow. Nevertheless, it should be pointed out that the combined zeolitic/mesoporous materials often have properties deviating from those of the zeolites and the amorphous mesoporous materials, depending on the applied synthesis method. This is due to the frequently occuring local differences in the active elements caused by differences in the extent of the zeolitic character as well as differences in diffusional behavior. A lot more research and in-depth characterization of the combined zeolitic/mesoporous materials is needed to fully understand their potential and the differences between these types of materials and their zeolitic and mesoporous counterparts.

**Table 3:** Comparison of the feasibility of the most important strategies for the synthesis of combined zeolitic/mesoporous materials (based on all the references used in this review).

approach	zeolitic character	level of mesoporosity	control over the mesopore structure	degree of interconnection	applicability to different types of zeolites and mesoporous materials	production costs

demetallation	high	medium	medium	low	medium–high	medium
recrystallization	medium	medium	medium	low	high	high
deposition	low	medium–high	high	low	high	high
delamination	high	low	low	low	low	high
hard templating	high	high	high	low–high	high	high–medium
soft templating	low–medium	high	medium–high	low–medium	high	high
template-free	low–medium	medium	low	medium	medium	medium
self-formation	—^a^	—^a^	low	—^a^	—^a^	medium

^a^Insufficient knowledge/examples/studies reported to make an assessment.

### 2 Catalysis and Ti-containing siliceous materials

Proper control of the synthesis parameters, elucidation of the formation mechanism and thorough characterization of nanoporous materials are essential to lead to the successful implementation of nanoporous materials in (industrial) chemical processes. The field in which nanoporous materials are applied most frequently is in heterogeneous catalysis. Therefore, in the next few paragraphs, the use of Ti-containing siliceous materials as catalysts will be discussed. Focus is particularly put on the already existing industrial applications and on those (lab-scale) applications with a high potential.

#### 2.1 Link between catalysis and Ti-coordination

**Figure 9:**
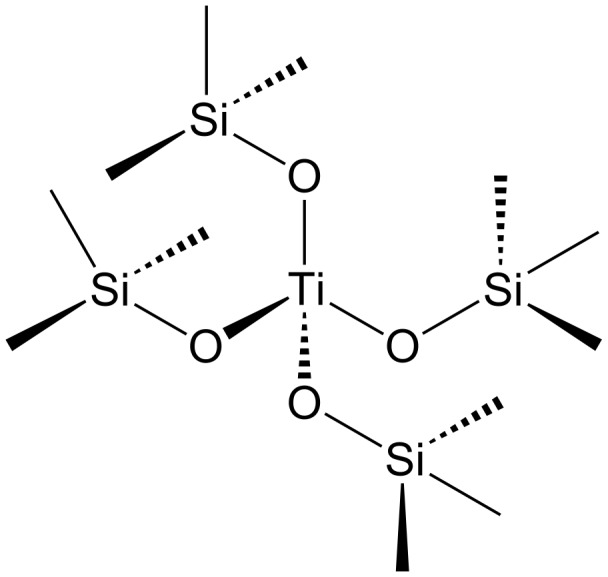
Isolated, tetrahedrally coordinated Ti(IV) site. Reprinted from [168]. Copyright 2008, with permission from Elsevier.

In the specific case of Ti-containing materials, the catalytic activity is ascribed to the presence of Ti(IV) in its structure [157]. These Ti(IV) sites are considered as redox-active centers [11,158]. Therefore, the typical reactions that can be catalyzed by Ti-containing materials are reactions in which electron exchange plays a key role, i.e., oxidation and reduction reactions [157]. Next to the amount of Ti present in the nanoporous materials, an equally important aspect that will have a large influence on the catalysis is the Ti coordination. Table 4 gives an overview of the different techniques that can be applied to determine the coordination. TiO*_x_* species can be present in a number of different coordinations, namely 4-, 5- and 6-fold [157]. Among these configurations, the isolated, 4-fold or tetrahedrally coordinated form is the most preferred one since this means that Si is perfectly isomorphically substituted by Ti in the structure, like in the full-grown zeolite TS-1 (Figure 9). This specific coordination gives rise to a high, and often unique, catalytic activity in oxidation processes (see further). A 5- and 6-fold coordination is often less wanted, since these species tend to form clusters (oligomerization) [167] and are correlated with defect sites in the titanosilicate structure [168]. When this oligomerization process goes even further, small TiO_2_ (crystalline anatase) particles can occur as extra-framework material, which is not built in the structure. Although crystalline TiO_2_ particles are well-known for their interesting semiconductor properties and their photocatalytic activity in photodegradation processes [169], TiO_2_ formation often needs to be avoided in the synthesis of Ti-containing nanoporous siliceous materials since its presence can be detrimental for the catalytic activity associated with tetrahedrally coordinated Ti [170–171].

**Table 4:** Overview of characterization techniques for the determination of Ti-coordination.

technique	references

UV–vis diffuse reflectance spectroscopy	[159]
photoluminescence IR spectroscopy	[160]
X-ray absorption spectroscopy (e.g., EXAFS, XANES, etc.)	[161–163]
vibrational spectroscopy (infrared and Raman)	[164–165]
electron paramagnetic resonance	[166]

#### 2.2 Redox catalysis

**Figure 10:**
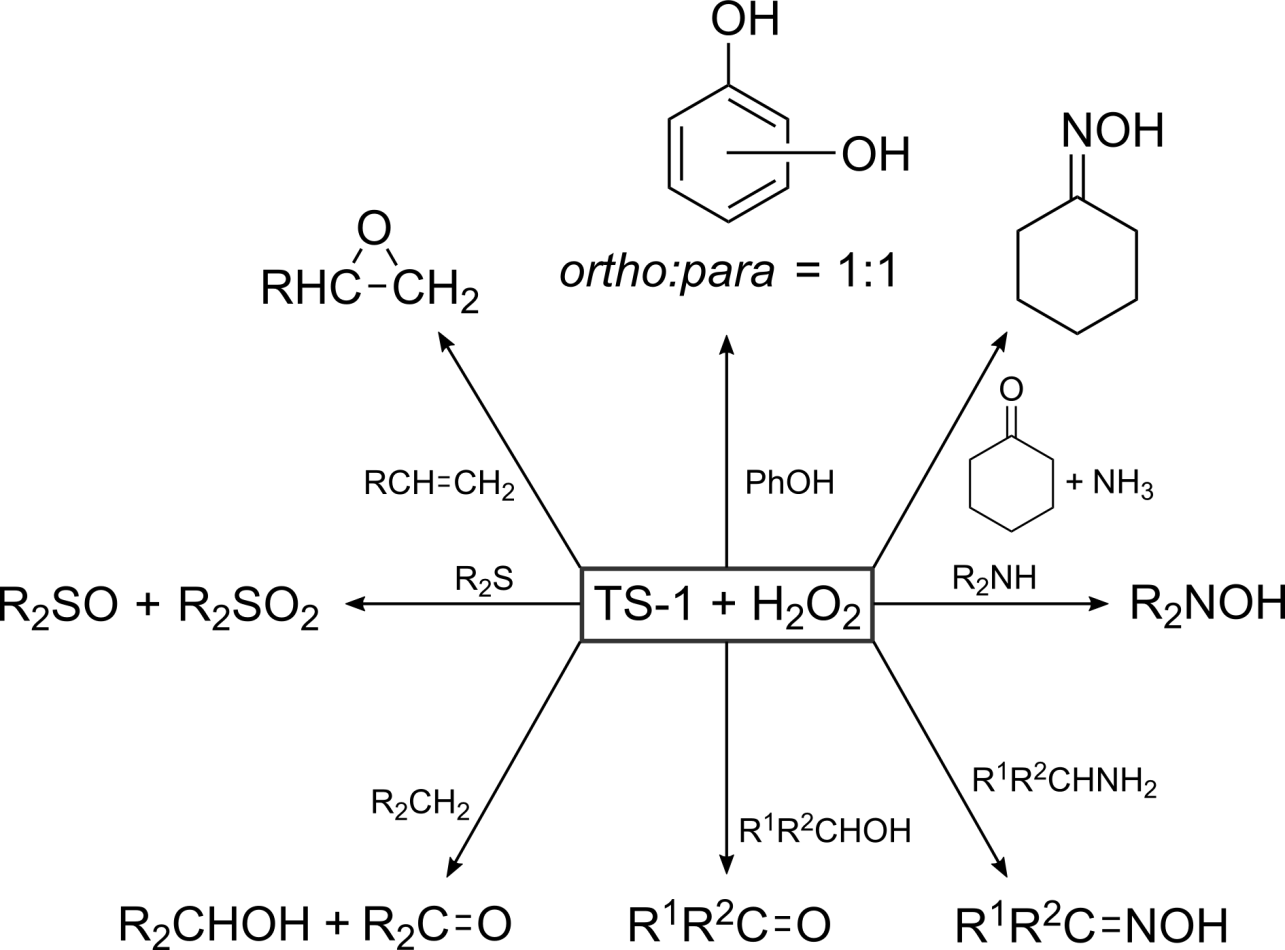
Schematic overview of the versatility of TS-1 as redox catalyst [172]. Reproduced with permission of Russian Chemical Reviews.

**Figure 11:**
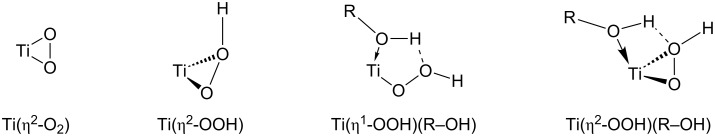
Different oxo-titanium species in TS-1. R–OH is a co-adsorbed alcohol molecule, stabilizing the hydroperoxide complex.

**Titanium-silicalite-1 – A versatile redox catalyst:** Titanium-silicalite-1 is a textbook example of a successful heterogeneous catalyst. This zeolite is considered as one of the most versatile redox catalysts available [157,172]. TS-1 currently finds application in various oxidation processes with H_2_O_2_ as oxidant (Figure 10), such as the epoxidation of alkenes [173], hydroxylation of aromatics [174–176], cyclization reactions [177], oxidation of alcohols [178–179] and ammoximation of ketones [180]. The strength of TS-1 as catalyst is attributed to (i) its shape-selectivity; (ii) its hydrophobic nature, enabling the preferential adsorption of the hydrophobic substrates also in the presence of water and (iii) its isolated, tetrahedrally coordinated Ti sites, preventing the undesired decomposition of H_2_O_2_ [181]. The active species in all the oxidation processes are believed to be an oxo-titanium complex formed by the interaction of H_2_O_2_ with Ti ions [162,181]. However, there is still no consensus on the exact structure of the Ti-peroxide complex in TS-1 (Figure 11).

Three processes have been industrialized over the past years, and this clearly reflects the importance of TS-1 for oxidation catalysis (the three upper reactions in Figure 10) [171–172,182–183]:

1. Hydroxylation of phenol: This reaction occurs in aqueous or aqueous-organic medium producing a mixture of hydroquinone (*p*-dihydroxybenzene) and catechol (*o*-dihydroxybenzene), which can be used as a reducing agent and precursor in fine chemistry. Water and tarlike compounds are the major byproducts. Conversion and selectivity are significantly higher than those achieved by acidic and radical catalysts. Therefore, since 1986, a plant near Ravenna, Italy, is producing 10,000 tons per year of diphenols with the aid of TS-1 as catalyst.
2. Ammoximation of cyclohexanone: This reaction is of major interest as cyclohexanone oxime is the intermediate in the manufacturing of caprolactam, the monomer for nylon 6. Conversion and selectivity of cyclohexanone to oxime is over 99% and the yield based on H_2_O_2_ is over 90%. Both Enichem as well as Sumitomo Chemical Co. operate such a TS-1 based caprolactam plant.
3. Epoxidation of propylene: Propylene oxide is one of the largest propene derivatives in production, ranking second behind polypropylene, and is primarily used as a reactive chemical intermediate. The “older” synthesis processes, such as the chlorohydrin route, generate a huge amount of byproducts, for example, for each ton of propylene oxide, 2 t of CaCl_2_ is obtained. In addition, 1.4 t of chlorine, 1.0 t of Ca(OH)_2_ and a large excess of water are needed. However, recently, BASF/Dow and Degussa-Evonik/Headwaters developed a new technology based on the use of aqueous solutions of H_2_O_2_ and a fixed-bed TS-1 reactor. At the end of 2008, the largest plant using this new process was started up in Antwerp by BASF/Dow.

Although the implementation of TS-1 has been successful, there are still some challenges left. Firstly, the use of H_2_O_2_ as oxidant is rather costly, which requires a low catalyst cost and very high process performance in order to meet economic targets. Therefore, researchers have been studying the possibility of H_2_O_2_ generation in situ, from H_2_ and O_2_ [182]. Secondly, like all zeolites, TS-1 suffers from accessibility and diffusion limitation problems for large and bulky molecules. For example, molecules with a kinetic diameter equal to or larger than cyclohexene are practically excluded from epoxidation reactions with TS-1 [85,184]. In general, high-value fine chemicals are usually too large to fit into the pores of the MFI structure of TS-1. This is one of the driving forces to synthesize larger pore titanosilicates.

**Large-pore Ti-containing siliceous materials:** As anticipated, larger pore Ti-activated siliceous materials, such as Ti-MCM-41 and Ti-SBA-15, are indeed able to catalyze oxidation reactions with bulky reactants [85,157,167,172,185–186]. Especially in epoxidations with large alkenes, such as limonene [167,186], mesoporous materials give rise to much higher activities than TS-1. However, although these mesoporous materials have clear advantages, they also suffer some major drawbacks: (i) They are less stable than TS-1, and especially the hydrothermal stability is significantly lower, and (ii) their hydrophilic nature, which is caused by their amorphous structure, leads to a lower catalytic activity [183,187]. This hydrophilicity will prevent efficient adsorption of the nonpolar/organic reactants, since the mesoporous materials show more affinity for water (from the aqueous H_2_O_2_ solution). By using an organic peroxide, such as *t*-butylhydroperoxide (TBHP), as oxidant, the catalytic activities can be remarkably improved [188]. Likewise, silylation of the surface of mesoporous materials, resulting in a more hydrophobic structure, is also a solution in many cases [189]. However, this does not solve the stability problems.

Combined zeolitic/mesoporous materials are expected to help to overcome these stability and hydrophilicity issues. Some successful examples have already been reported in the literature [51–52,58–59,78–79,82,122]. For example, MTS-9 [52] shows a high hydrothermal stability (over 120 hours in boiling water) and a high oxidation ability for small (phenol and styrene) as well as larger molecules (2,3,6-trimethylphenol) with 30% aqueous H_2_O_2_ as oxidant. As mentioned above, this material is formed in a two-pot templating strategy, wherein preformed, nanosized titanosilicate precursors are combined with polymer surfactants. Its high activity has been ascribed to the TS-1-like Ti-species present in MTS-9 and the fact that its relatively thick pore walls (4.8 nm) contain primary and secondary structural building units, similar to TS-1. Another nice example is Ti-MMM-2 [122], a one-pot templated material with a microporous TS-1 phase and a mesoporous Ti-MCM-48 phase. This material shows a higher activity (61%) than both TS-1 (16%) and Ti-MCM-48 (42%) for the epoxidation of cyclohexene with TBHP (in decane) as oxidant. Although the difference in catalytic activity was compared to the purely mesoporous material and the zeolite, no data concerning the stability are reported, nor data with H_2_O_2_ as oxidant. The fact that an organic peroxide is used as oxidant instead of H_2_O_2_, meaning a more hydrophobic medium instead of a hydrophilic one, can by itself lead to differences in the catalytic performance of nanoporous materials (as mentioned above for the purely mesoporous materials). Reichinger et al. [59] investigated the effect of the reaction medium by performing the cyclohexene epoxidation in both a hydrophobic and a hydrophilic environment. The studied material was a mesoporous structure assembled from TS-1 nanoparticles. This material outperformed TS-1 and Ti-MCM-41 in the cyclohexene epoxidation, both under hydrophobic conditions (TBHP and decane) and under hydrophilic conditions (H_2_O_2_ and water/CH_3_OH). However, in the epoxidation of the smaller molecule 1-hexene, the (more hydrophilic) combined zeolitic/mesoporous material failed completely in the hydrophilic medium whereas the hydrophobic TS-1 zeolite showed a high catalytic activity. These experiments perfectly exemplify the need for (i) a suitable combination of the reagents and the pore dimensions of the catalyst and (ii) the importance of compatibility between the characteristics of the materials and the reactions conditions (e.g., hydrophilicity). Here, also no information on the stability of the combined materials was given. On the other hand, not all combined zeolitic/mesoporous materials outperform their purely zeolitic or mesoporous counterparts. We recently studied three types of combined zeolitic/mesoporous materials in the epoxidation of cyclohexene with H_2_O_2_ [85]. Here, two materials were obtained by postsynthetic deposition of TS-1 nanoparticles on SBA-15 (SBA-TS-15-pH 1 and SBA-TS-15-pH 13) and one structure was formed by a one-pot templating approach (meso-TSM). Both SBA-TS-15 materials showed similar (low) activity as compared to TS-1, while the performance of meso-TSM lay in between TS-1 and Ti-MCM-41. In the case of meso-TSM, combined zeolitic/mesoporous materials did not give rise to an enhanced performance in comparison with their purely mesoporous counterparts. Also Chenevieve et al. [60] observed that their titanosilicates with a mesoporous or microporous hierarchical structure did not possess the superior catalytic properties expected for hierarchical catalysts: In particular, the gain in diffusion properties was totally inhibited by the increase in hydrophilic character of the material.

Notice that divergent catalytic behavior is observed, which highly depends on the properties of the materials and on the synthesis method for the formation of the titanosilicates. Moreover, important information on these Ti-containing combined zeolitic/mesoporous materials concerning stability, regenerability and scaling up is often lacking or not yet investigated. Therefore, more research concerning their structural properties and catalytic behavior is necessary to fully explore and understand the catalytic potential of the combined zeolitic/mesoporous materials. At the moment, it is still too early to tell whether industrialization of these combined zeolitic/mesoporous materials as redox catalysts can be expected in the near future. However, major progress is to be expected in this area of research.

## Conclusion

This review highlighted the most common synthesis approaches for the formation of combined zeolitic/mesoporous materials. These materials have been gaining a lot of interest during the last decade, since they are an attempt to combine the superior properties of zeolites (high stability, catalytic activity and selectivity) with those of mesoporous materials (improved diffusion and accessibility for larger molecules and viscous fluids). Some of the synthesis strategies have been extrapolated from the synthesis of purely mesoporous materials (e.g., the two-pot templating approach) whereas others have been newly developed or specifically designed for the formation of combined zeolitic/mesoporous materials (e.g., one-pot templating approach with organosilane surfactants and postsynthetic routes). All synthesis methods have their advantages and disadvantages, meaning that there is no obviously superior synthesis strategy. Therefore, the applied synthesis approach should depend on the final application, the desired properties of the materials and the resources available. Moreover, a lot more research on the combined zeolitic/mesoporous materials is needed to fully understand the discrepancies between these types of materials and their zeolitic and mesoporous counterparts. With regards to the Ti-containing combined zeolitic/mesoporous materials as redox catalysts, a lot of progress has been made in recent years with the development of interesting materials, especially for oxidation reactions of bulky molecules. However, sufficient information on the local structural properties and diffusion behavior of the various synthesized materials in relation to the synthesis methodology is still lacking. In addition, important information concerning the stability, regenerability and scaling up of these combined materials is still missing, making it difficult to draw a conclusion on their potential (industrial) implementation. Therefore, further investigation is necessary in order to fully explore the (catalytic) potential of the combined zeolitic/mesoporous materials.
